# The Efficacy of the Low-FODMAP (Fermentable Oligosaccharides, Disaccharides, Monosaccharides, and Polyols) Diet in Irritable Bowel Syndrome: A Systematic Review and Meta-Analysis

**DOI:** 10.7759/cureus.77053

**Published:** 2025-01-07

**Authors:** Zahid Khan, Syed Aun Muhammad, Mehul S Amin, Amresh Gul

**Affiliations:** 1 Cardiology, University of South Wales, Pontypridd, GBR; 2 Cardiology, University of Buckingham, Buckingham, GBR; 3 Cardiology, Barts Heart Centre, London, GBR; 4 Cardiology, Mid and South Essex NHS Foundation Trust, Southend-on-Sea, GBR; 5 Internal Medicine, Southend University Hospital, Southend-on-Sea, GBR; 6 General Practice, GP Clinic, Brisbane, AUS

**Keywords:** british dietetic association, general dietary advice, high-fodmap diet, ibs (irritable bowel syndrome), ibs-sss, irritable bowel syndrome with diarrhea, low-fodmap diet, medical subject headings, structural individual low fodmap dietary advice, treatment of irritable bowel syndrome

## Abstract

Irritable bowel syndrome (IBS) is frequently observed in clinical practice and affects people from different parts of the world. The pathogenesis and aetiology are not well-defined or fully understood; however, altered bowel movements, psychological factors, and visceral hypersensitivity may contribute to symptoms via a pathway mediated by serotonin and other enteric neurotransmitters. Altered bowel movements, including diarrhoea and constipation, abdominal pain relieved by passing flatus, and bloating are the main salient features of this condition. This systematic review and meta-analysis aimed to determine the effectiveness and efficacy of a low-fermentable oligosaccharides, disaccharides, monosaccharides, and polyols (low-FODMAP) diet in these patients. Systematic searches were conducted on PubMed, Medline, Google Scholar, and Cochrane Library. Randomised controlled trials (RCTs), systematic trials and cohort studies that included keywords about IBS and a low-FODMAP diet were included. Exclusion criteria included studies that were not in the English language, not relevant to IBS, diet-related to inflammatory bowel disease, or not pertinent to the subject. A total of 41 studies were included in this systematic review and meta-analysis. There was significant heterogeneity among the RCTs; hence, a random-effects model was used. The systematic review included a total of 8460 patients across 36 studies, with follow-up durations ranging from 11 to 16 months. Specifically, the meta-analysis included 15 RCTs with 1118 participants and follow-up durations from two days to nine weeks and six cohort studies including 292 patients with follow-up durations from two weeks to two years. The risk ratio (RR) was 1.21 (95% confidence interval= 0.98-1.51), and the I^2^ value ​​​​​was 63% for global symptom improvement with a low-FODMAP diet using a random-effects model. There was a low risk of bias in the RCTs. Five studies were included evaluating the effect of a low-FODMAP diet on quality of life, and these studies did not show any statistically significant benefit of a low-FODMAP diet on quality of life, although a mean difference of 4.59 (95% CI 1.50-7.67) was observed. The risk of bias was moderate to severe in the observational studies included in this review. Food intolerance is increasingly recognised as a contributory factor in IBS, and its role in the pathogenesis and precipitation of symptoms is being explored. Specific mechanisms include the fermentation of FODMAPs by the gut microbiota, leading to gas production and subsequent symptoms.

## Introduction and background

Irritable bowel syndrome (IBS) is one of the most prevalent chronic functional gastrointestinal syndromes worldwide. In some developed countries, the prevalence is higher in women than in men. The diagnosis of IBS is based on the ROME IV criteria, the inclusion of various symptoms such as altered bowel habits, including constipation and diarrhoea, abdominal pain/discomfort improved on passing flatus/defecation, and the absence of organic causes, such as inflammatory bowel disease (IBD), celiac disease, and colon cancer [1]. IBS symptoms must have occurred six months before the initial presentation, along with clinical features. IBS is usually divided into subcategories according to the predominant symptoms: IBS with diarrhoea (IBS-D), IBS with constipation (IBS-C), and IBS mixed (IBS-M) [2]. Psychological factors play a significant role in precipitation, persistence, decision to seek treatment, and response to treatment [3-5].

Food intolerance is increasingly recognized as a contributory factor in IBS, and its role in the pathogenesis and precipitation of symptoms is being explored. Specific mechanisms include the fermentation of FODMAPs by the gut microbiota, leading to gas production and subsequent symptoms. Food intolerance is generally not the cause of IBS but is a trigger for symptoms. Common triggers of food intolerance include caffeine, alcohol, carbonated drinks, fatty foods, fibre, lactose-containing foods, wheat, and spicy food [6-9]. However, avoidance of gluten-free and certain foods may make patients susceptible to long-term nutritional deficiencies and low body weight [6,7]. Therefore, nutritional counselling from a dietitian is important when implementing long-term dietary restrictions.

Food, including the fermentable oligosaccharides, disaccharides, monosaccharides, and polyols (FODMAP) diet, is often considered an aggravating factor for IBS symptoms. In recent years, there has been growing interest in FODMAP, which is found in many common foods. Examples of FODMAPs include fructose, lactose, sugar alcohols (sorbitol, maltitol, mannitol, xylitol, and isomalt), fructans, and galactans, which are present in a wide range of foods, such as wheat, rye, vegetables, fruits, and legumes [8-13]. Dietary interventions have been claimed to result in symptomatic improvement in both acute and chronic phases of IBD. By altering the microbiome, metabolome, host barrier function, and innate immunity, disease progression can be mitigated, and potentially fatal complications can be avoided. Studies have shown that a semi-vegetarian diet has protective effects, including relieving symptoms, slowing disease progression, improving the quality of life, and preventing the recurrence of celiac disease.

Traditionally, the treatment of IBS has focused on medications such as laxatives, anticonvulsants, and antidepressants. However, in recent years, attention has been focused on dietary interventions and cognitive/emotional therapies [10-15]. A diet low in FODMAP has received increasing attention and has been proposed as a first-line nutritional therapy for the management of IBS.

**Figure 1:**
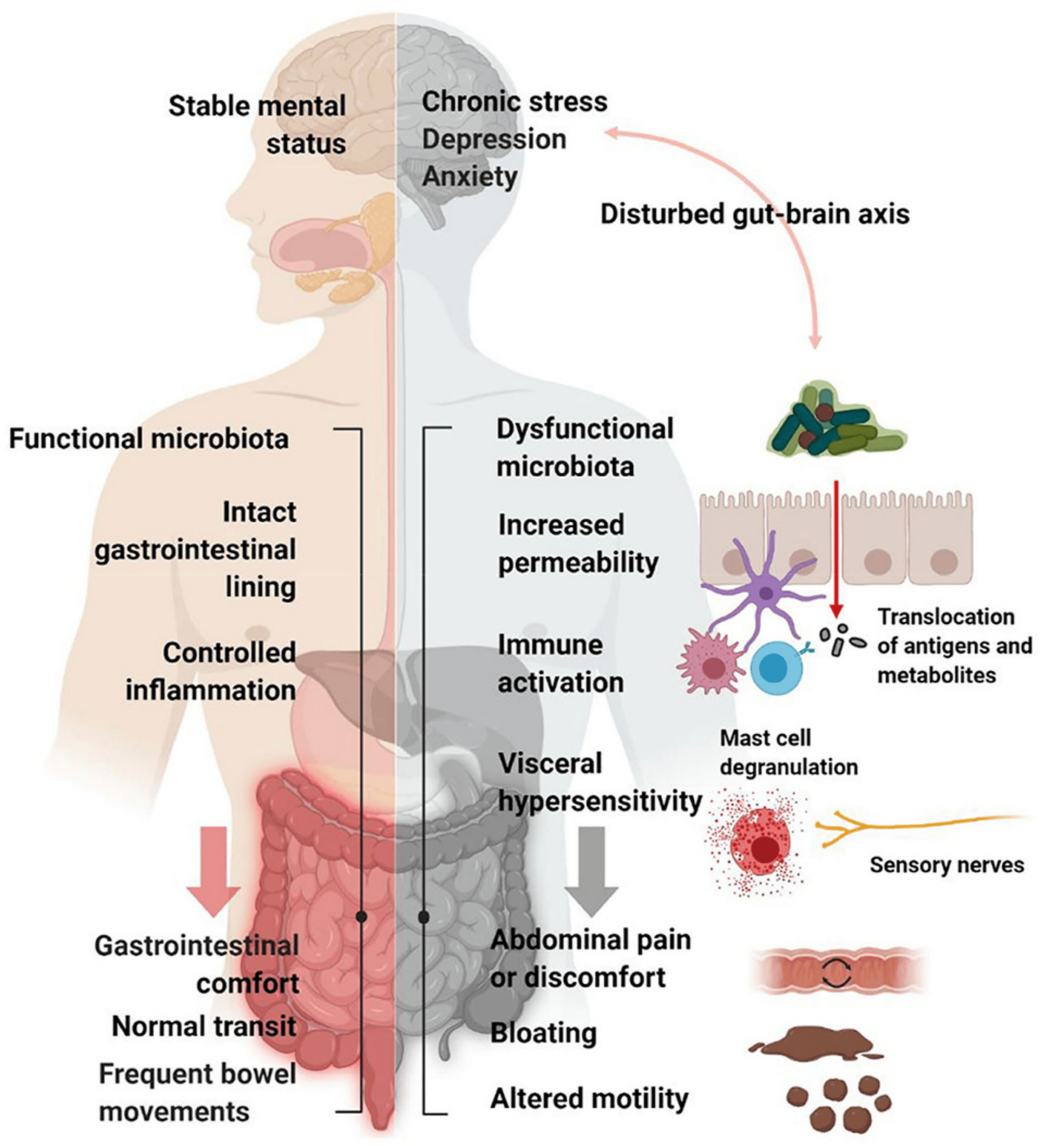
Schematic representation of IBS pathophysiology and various factors considered to be involved in the generation of IBS symptoms. Permission was obtained from the authors for the reproduction and reuse of this figure [17]. IBS: Irritable bowel syndrome

Several randomised controlled trials (RCTs) have been conducted to determine the efficacy and effectiveness of the FODMAP diet; however, the findings from most of these studies are mixed [12-17]. Therefore, this systematic review aimed to determine the effectiveness of a low-FODMAP diet in IBS patients’ symptoms by summarising and analysing the findings of these trials. Figure 1 shows a schematic representation of IBS pathophysiology and the various factors considered to be involved in the generation of symptoms in patients with IBS.

## Review

Materials and methods

Search Strategy

The study was registered with PROSPERO and the National Institute of Health Research under the registration number CRD42023428265. A systematic review was conducted following the preferred reporting items for systematic reviews and meta-analyses (PRISMA) guidelines, utilising multiple search engines to establish research on the effectiveness of the low-FODMAP diet in irritable bowel syndrome patients. The present systematic review used the population, intervention, comparator, and outcomes (PICO) model to define the search question. This review aimed to assess the efficacy of a low-FODMAP diet in improving the symptoms of patients with irritable bowel syndrome.

We conducted a literature search using the following four databases: MEDLINE, Google Scholar, Cochrane Library, and PubMed. The Medical Subject Headings (MeSH) used for the search were: “Irritable Bowel Syndrome” [MeSH Terms], “Low FODMAP Diet” [MeSH Terms], and combinations of the two, and also included text keywords “IBS”, “Irritable Bowel Syndrome”, and “Low FODMAP diet”. A total of 2729 articles were identified, of which 36 were included in this systematic review and meta-analysis [1-51]. A literature search was performed by two researchers from January 2024 to February 2024. Any disagreements were resolved through mutual agreement and input from a third researcher. The data extracted from these studies were exported to an electronic Google sheet accessible to all researchers involved in this review. Titles and abstracts of all identified articles were screened by two independent reviewers. Discrepancies were resolved by discussion with a third reviewer. A standardised data extraction form was utilized. The key data extracted included demographic details of the patients, type of study, author details and publication year, number of patients in each group, type of intervention in the control and intervention groups, follow-up duration, and outcome of interest.

Eligibility Criteria

RCTs (studies published in English) were selected for inclusion in the present systematic review if they reported research conducted in adult patients (> 18 years old) with IBS. RCTs, systematic reviews, and cohort studies were included if they provided complete data. Most of the identified articles (2154) were excluded because although they were included in the title diet and IBD, they were not considered relevant to this study due to the different outcomes assessed. After the initial screening of the titles, abstracts were analysed, and articles were excluded if their aims and objectives were not considered relevant. Of the 205 studies, five were not retrieved, 10 did not use any assessment tool, and 154 did not assess the role of a low-FODMAP diet in IBS or had a different outcome and were excluded. The remaining 36 studies were included in the systematic review, and 15 studies were included in the meta-analysis. 

Exclusion Criteria

Studies involving patients younger than 18 years of age or patients with a combination of IBS and IBD were excluded. Articles not written in the English language were excluded due to resource constraints for translation and bias risk from potential translation errors. Articles reporting other study types (commentaries, individual opinions, case reports, and case series), and those with missing data were also excluded. Articles that were not freely available were excluded. Studies published before 2015 were excluded from the review.

Inclusion Criteria

Studies involving adult patients aged > 18 years, studies involving patients with IBS published in English, and studies published after 2010 were included in this review to focus on the latest research. Only systematic reviews, RCTs, and observational studies were included in this meta-analysis. 

**Figure 2:**
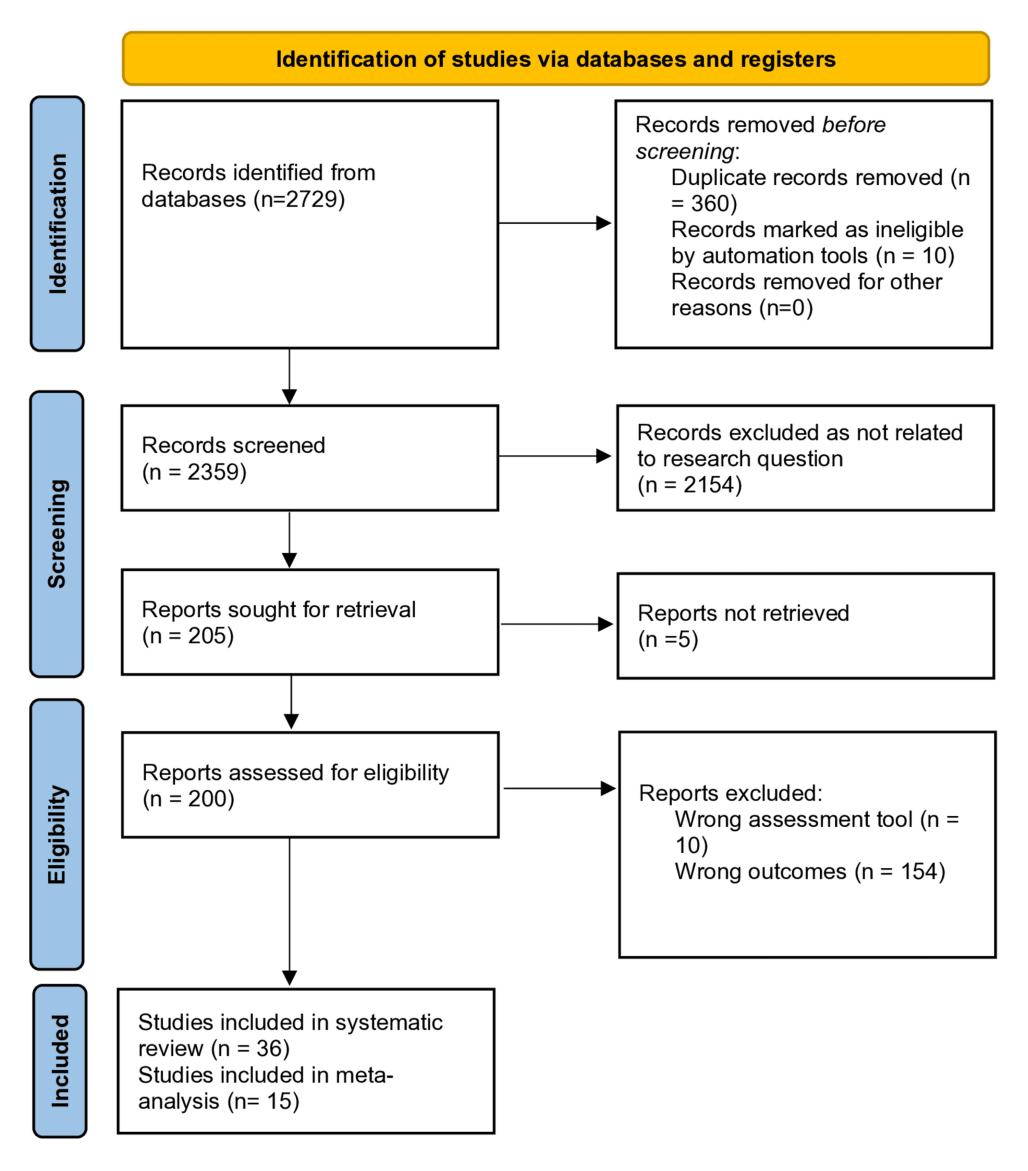
Preferred Reporting Items for Systematic Reviews and Meta-Analyses (PRISMA) chart showing the literature methodology.

A further breakdown of the included studies and literature search is presented in the Preferred Reporting Items for Systematic Reviews and Meta-Analyses (PRISMA) chart (Figure 2). The Population, Intervention, Comparison, and Outcome (PICO) table is presented in Table 1.

**Table 1:** Population, Intervention, Comparison, and Outcome table. PICO: Population, Intervention, Comparison, and Outcome; LOW-FODMAP: Low-Fermentable oligosaccharides, disaccharides, monosaccharides, and polyols; NICE: National Institute for Health and Care Excellence; IBS: Irritable bowel syndrome

Variable	Definition
Population	Adult patients with IBS, patients with IBS and without IBD, patients not receiving medical therapy for IBS
Intervention	A low-FODMAP diet
Comparison/control	High-FODMAP Diet, NICE diet, low lactose diet, fructose-restricted diet, British Dietetic Association diet, structural individual low-FODMAP dietary advice, typical American childhood diet
Outcome	Global symptom improvement such as abdominal pain, bloating, stool consistency and frequency, quality of life

Data Synthesis and Statistical Analysis

**Figure 3:**
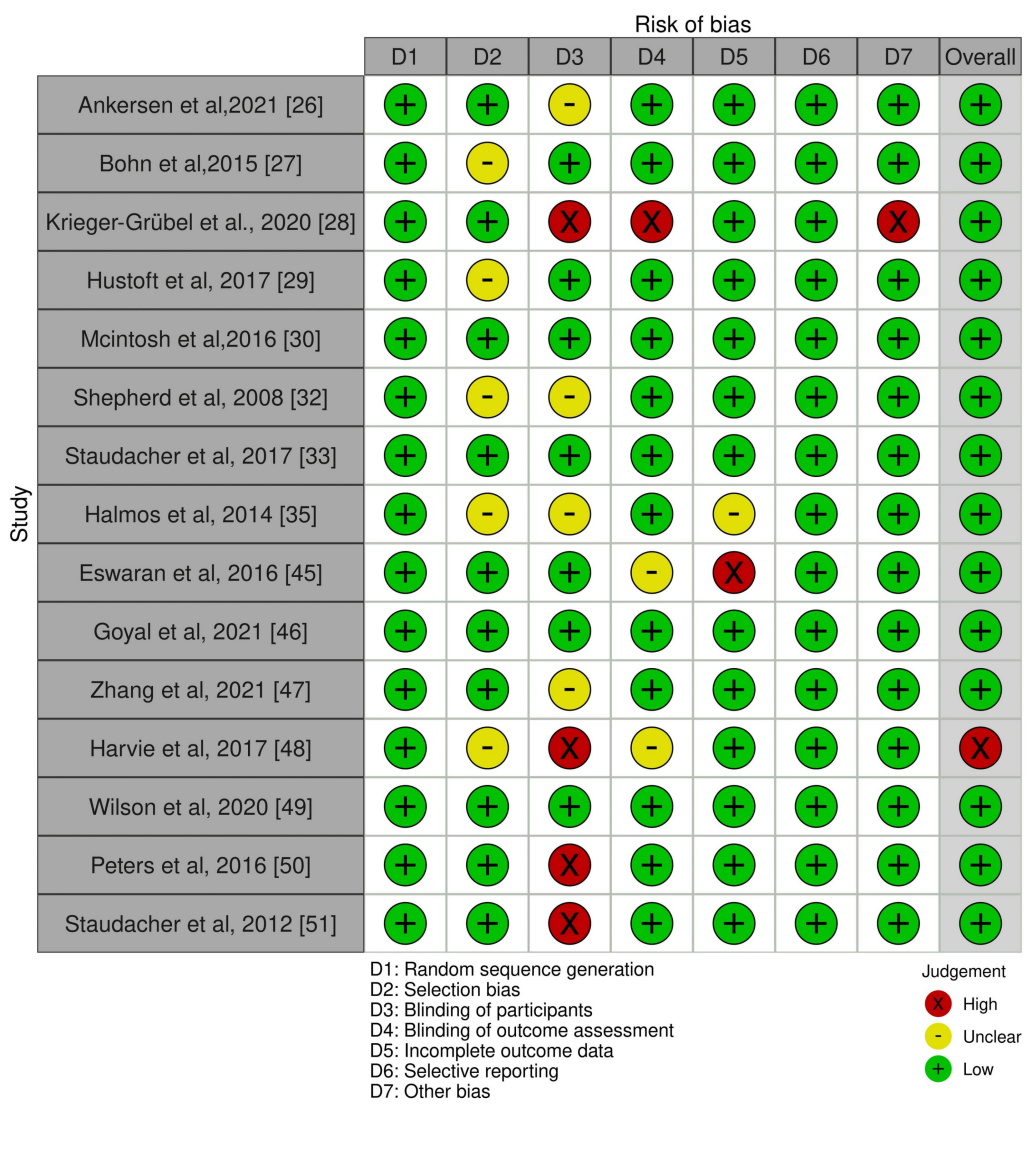
The risk of bias assessment traffic light chart for randomised controlled trials included in the meta-analysis.

**Figure 4:**
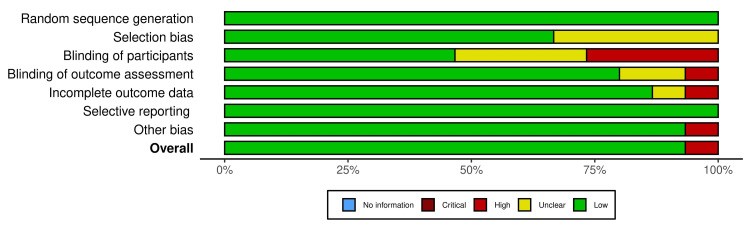
The risk of bias plot for randomised controlled trials included in the meta-analysis showing risk of bias by percentage.

**Figure 5:**
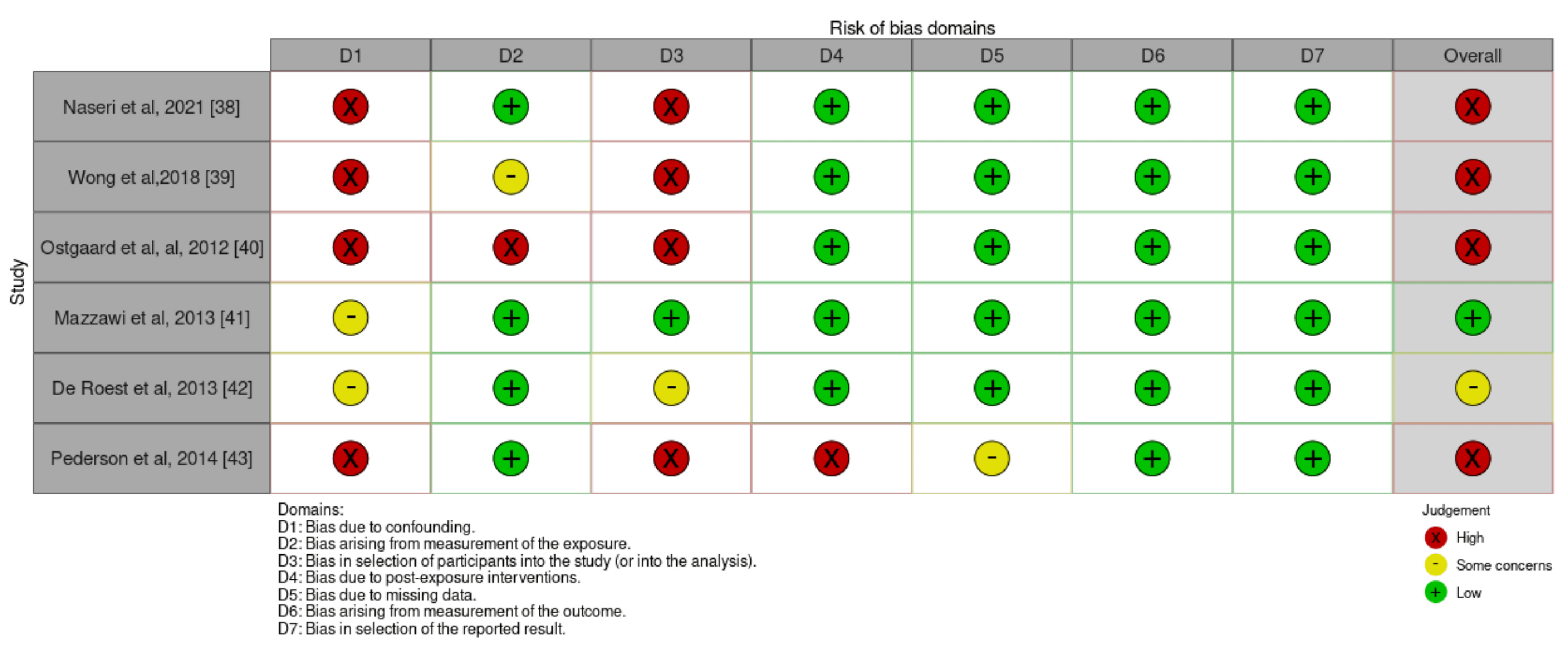
Risk of bias assessment traffic light chart using ROBINS-E for observational studies showing moderate to high risk of bias. ROBINS-E: Risk Of Bias In Non-randomized Studies - of Interventions

**Figure 6:**
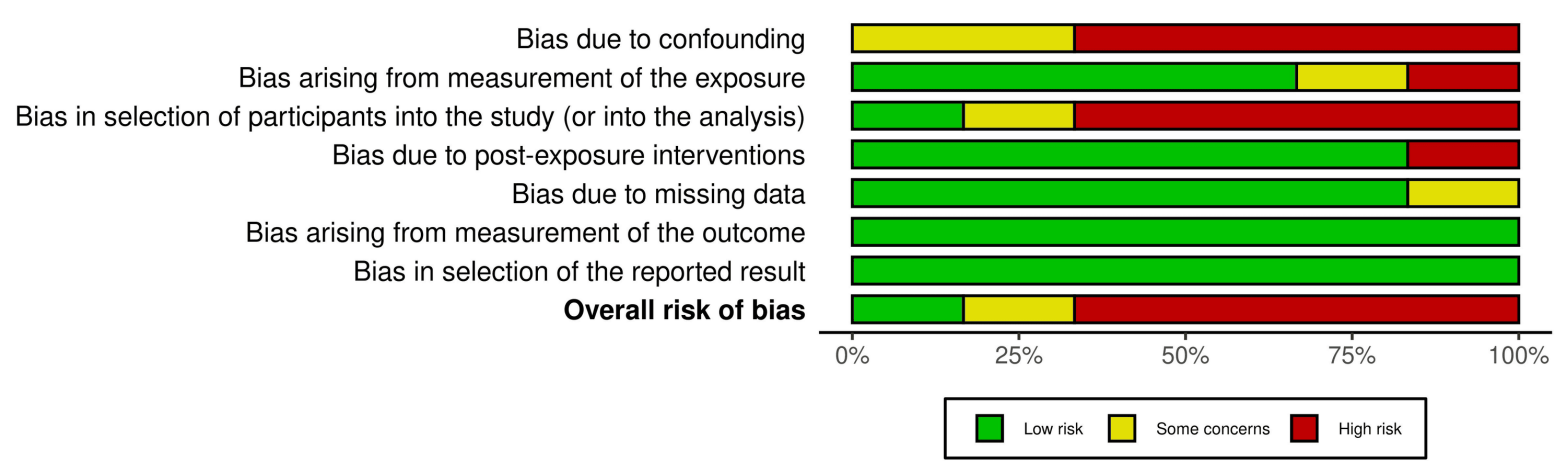
The risk of bias assessment by percentages for the observational studies included in this review.

Data analysis was performed using RevMan 5 (version 5.3, Cochrane Collaboration), and the odds ratios (ORs) were calculated with 95% confidence intervals (CIs) for global symptom improvement in IBS patients in the LFMD group compared with the control group. A random-effects model was used to evaluate heterogeneity, and an I^2^ value >50% was considered to indicate significant heterogeneity. Forest plots with OR were used as the primary outcomes. The revised Cochrane risk-of-bias tool (ROB 2) and Risk Of Bias In Non-randomized Studies - of Interventions (ROBINS-E) were used to assess the risk of bias in RCTs and observational studies, respectively. The risk of bias assessment was low in RCTs; however, the risk ranged from moderate to high in observational studies (Figures 3-6).

Results

Study Selection and Search Results

After applying the inclusion and exclusion criteria outlined above, 11 systemic reviews were identified involving patients with IBS and a low-FODMAP diet. Additionally, 19 RCTs and six cohort studies were identified [1-51]. The results of the systematic reviews are presented in Table 2.

**Table 2:** Demographic data for the systematic reviews included in this study. FODMAP: Fermentable oligosaccharide, disaccharide, monosaccharide and polyol; IBS-C: Irritable bowel syndrome-constipation; CI: Confidence interval; SMD: Standard mean difference; OR: Odds ratio; RR: Relative risk; BDA/NICE: British Dietetic Association/National Institute for Health and Care Excellence

Author	Methodology	Number of trials assessed	Number of participants	Duration	Conclusion
Wang et al., 2021 [1]	Symptom improvement in patients following a low-FODMAP diet compared to control groups	10	511	11 days to 9 months	Patients following a low-FODMAP diet had improvement in symptoms compared to control groups (n=420, risk ratio = 1.54)
Altobelli et al., 2017 [12]	Comparison of the low-FODMAP diet to either a traditional IBS diet or a high-FODMAP diet	12 meta-analyses, six RCTs and six cohort studies	887 patients completed the studies.	3 weeks to 36 weeks	Patients with low-FODMAP diets had better symptom control, abdominal pain and bloating than those following a traditional IBS diet or medium/high FODMAP diet. There was no difference in the stool consistency between the groups.
Schumann et al., 2018 [14]	Impact on symptoms among patients with IBS – low-FODMAP diet vs various diets	9	596	2 days to 16 months	Significant improvement in symptoms among low-FODMAP cohorts compared to other diets. LFD was more effective for short-term symptom relief compared to the control group on health-related quality of life (SMD = 0.36; 95% CI = 0.10–0.62; P = 0.007).
Staudacher et al., 2017 [18]	Examined trials that evaluated the effect of low-FODMAP diets on quality of life through various IBS symptom-scoring scores	10	685	4 weeks to 3 months	9 out of 10 trials demonstrated a reduction in symptoms of IBS among those with low-FODMAP diets. The study by Staudacher et al. did not demonstrate any significant difference in the primary outcome between the two groups.
Black et al., 2021 [19]	Compared low-FODMAP diets to habitual diets, high FODMAP diets and BDA/NICE dietary advice	13	944	4 weeks to 3 months	A low-FODMAP diet was superior to all other forms of diet, including habitual diet and BDA/NICE dietary advice in improving IBS symptoms such as abdominal pain severity, abdominal bloating and severity but was not superior for bowel habit to the other interventions.
Krogsgaard et al., 2017 [20]	The impact of low-FODMAP diets on symptoms in patients with IBS was assessed compared to controls	9	542	3 weeks to 3 months	A low-FODMAP diet had a positive impact in 5 out of the 9 trials, while it had an equivalent impact on symptoms in 4 out of the 9 trials when compared to the control.
Manning et al., 2020 [21]	Comparison of low-FODMAP diet to a variety of either other diets, non-dietary interventions or probiotics, observing the impact on symptoms in patients with IBS	10	840	2 days to 6 months	Patients on low-FODMAP diets had greater improvement in symptoms compared to all other diets, probiotics or non-dietary interventions.
Dionne et al., 2018 [22]	Symptom improvement in patients following low-FODMAP diet compared to control groups (part of the review examining gluten-free diet has been excluded)	7	397	3 weeks to 4 weeks	There was a significant improvement in symptoms in patients following a low-FODMAP diet compared to the control group (RR = 0.69). Three studies showed a low-FODMAP diet to be associated with reduced global IBS symptoms compared with alternative diets and one study showed that a low-FODMAP diet was superior to a high FODMAP diet in global IBS symptom reduction. Two studies showed the superiority of a low-FODMAP diet over a traditional diet in improving IBS symptoms.
Rao et al., 2015 [23]	Evaluation of fibre and low-FODAMP diets on IBS symptoms	6	311	3 weeks to 9 weeks	Significant improvement in all trials of symptoms among IBS patients following low-FODMAP diets. The low-FODMAP diet was associated with significant short-term IBS-C symptom improvement compared to a traditional diet.
Hahn et al., 2021 [24]	The effect of low-FODMAP diets on quality of life, stool habits and overall symptoms	22 (20 RCTs and two cohort studies)	682	2 weeks to 16 months	Significant improvement in patients on the low-FODMAP diet compared to control groups (OR, 2.14; 95% CI, 1.56, 2.93; I^2^ = 0%).
Turco et al., 2018 [25]	Impact on symptoms in patients with IBS following the low-FODMAP diet compared to control	13 intervention trials and 6	2065	1 day to 3 months	A low-FODMAP diet was beneficial in 12/13 intervention trials.

Eleven systematic reviews (Table 1) assessed low-FODMAPS diets against other forms of diet in patients with IBS. The studies showed that adherence to a low-FODMAPS diet produced a greater improvement in symptoms, such as abdominal pain and bloating, compared to other diets, including high/medium-FODMAPS diets, controls, and probiotics. 

The total number of patients in these studies was 8460, and the follow-up duration varied from 2 to 16 months. Wang et al. (2021) performed a systematic review based on 10 studies, including 511 patients. on a low-FODMAP diet in patients with IBS. They found global improvement in IBS symptoms, stool consistency, and frequency in patients on a low-FODMAP diet compared to those on a normal diet. Five of these ten studies assessed the global symptom change using IBS-SSS, showing a significant reduction in total IBS-SSS score, pain intensity and frequency, and dissatisfaction with bowel habit; however, no statistically significant effect was found on abdominal distension when comparing the low-FODMAP diet with normal diet in IBS patients [11]. Another study by Altobelli et al. (2017), comprising 887 patients from six RCTs and six cohort studies, found that a low-FODMAP diet was superior to the traditional IBS diet, and patients had a significant reduction in abdominal pain (OR 0.44) and bloating (OR 0.32) but limited efficacy in stool consistency (0.24) and stool frequency in those receiving a low-FODMAP diet. Upon comparing the low-FODMAP diet with the medium/high FODMAP diet, a similarly significant reduction in abdominal pain (OR 0.17) and bloating (OR 0.13) was observed. Six cohort studies also showed an improvement in abdominal pain and bloating from their baseline following low-FODMAP dietary intervention [12]. Staudacher et al. (2017) found that the short-term use of a low-FODMAP diet produced a 50-80% reduction in IBS symptoms in 10 RCTs evaluated [18]. Black et al., based on their study including 13 RCTs, reported that a low-FODMAP diet was associated with improvement in symptoms compared to sham dietary advice, NICE diet, and high FODMAP diet in patients with IBS [19]. Staudacher et al. (2017) showed greater odds of reduction in abdominal pain, bloating, and overall GI symptoms compared to the control, with reported odds ratios of 1.81, 1.75, and 1.81, respectively.

Manning et al., based on 10 studies using IBS-SSS to compare the severity of symptoms, showed that a low-FODMAP diet was superior in symptomatic improvement reported by participants compared to other dietary interventions including a high-FODMAP diet, sham diet, gluten-free diet, generalised healthy eating, and traditional advice. The improvement was particularly noticeable in IBS subtypes IBS-D and IBS-C when comparing a low-FODMAP diet to other dietary interventions [21]. Dionne et al. evaluated seven RCTs involving 397 patients on low-FODMAP diets against various diets and two RCTs on gluten-free diets in patients with IBS [22]. This study confirmed the findings of previous studies that a low-FODMAP diet was associated with reduced global symptoms compared with control patients. Patients on gluten-free diets also showed improved symptom control compared to those in the control group. In another study, based on six studies assessing the impact of a low-FODMAP diet on IBS symptoms, patients on a low-FODMAP diet showed significant improvement in symptoms such as abdominal pain, bloating, stool frequency, diarrhoea, and constipation. This study also highlighted the importance of long-term dietary restrictions, including the risk of deficiencies [23].

Hahn et al. analysed 20 studies, including 682 patients who were followed up to 16 months, and this study also supported the findings from previous studies showing symptomatic improvement in patients on a low-FODMAP diet [24]. It is important to recognise that, despite symptom improvement, this study did not demonstrate a significant difference in the quality of life between the two groups. Studies in this systematic review used different questionnaires to assess patients' symptoms, 13 studies used the IBS-SSS form, seven studies used the VAS or Likert scale form, and the rest only reported responses to an IBS adequate relief question. The effect of LFD on IBS symptom severity was reported in 20 studies. The LFD group showed a moderate reduction in IBS symptom severity compared with the control group (SMD -0.53; 95% CI, -0.68, -0.38; I²=39%). These studies also used different questionnaires to assess patients' symptoms, including the IBS-SSS form (13 studies), the VAS or Likert scale form (seven studies), and responses to an IBS adequate relief question. When analysing studies that reported both pre- and post-intervention IBS-SSS scores, a mean reduction of 52.6 points (95% CI, −76.48, −28.72; I^2^ = 66%) was observed in the LFD group. The number of patients who showed a reduction in IBS-SSS of more than 50 points and who reported adequate symptom relief in the LFD group compared to the control group. In the IBS-SSS subscale score analysis, the most substantial improvement occurred for “severity of abdominal distension” (SMD, −0.47; 95% CI −0..67, −0.27; I^2^ = 25%), followed by “dissatisfaction with bowel habits” (SMD, −0.43; 95% CI, −0.63, −0.23; I2 = 24%), “frequency of abdominal pain” (SMD, −0.30; 95% CI, −0.55, −0.06; I^2^ = 50%), and “severity of abdominal pain” (SMD, −0.26; 95% CI −0.47, −0.05; I^2^ = 32%). The IBS-QoL was analyzed in 11 studies and showed a slight numerical, but not statistically significant, improvement after LFD compared with the control intervention (SMD, 0.24; 95% CI, 0.02, 0.47; I² = 60%). There was significant heterogeneity between studies. Similarly, Schumann et al. performed a systematic review and meta-analysis of nine RCTs which showed a low-FODMAP diet compared with other diets and demonstrated improvements in gastrointestinal symptoms, abdominal pain, and health-related quality of life [25]. However, the long-term outcomes and safety require further investigation.

The results of the RCTs are pooled in Table 3.

**Table 3:** Demographic data and findings for randomised controlled trials included in this study. BDA: British Dietetic Association; NICE: National Institute for Health and Care Excellence; FODMAP: Fermentable oligosaccharides, disaccharides, monosaccharides, and polyols: OR: Odds ratio; CI: Confidence interval; LLD: Low lactose diet; BRD: Brief advice on a commonly recommended diet; SILFD: Structural individual low-FODMAP dietary advice; TACD: Typical American childhood diet

Author	Methodology	No. of participants	Study duration	Tools used to monitor success	Intervention group	Control group	Conclusion
Zahedi et al., 2018 [8]	Patients were randomised to either LFD or General Dietary Advice (GDA)	110 (55 patients were randomly assigned to the low-FODMAP diet and 55 patients were assigned to the BDA diet).	6 weeks	Improvement in symptom severity score	Patients on LFD had a greater reduction in symptoms than GDA (p<0.001)	Patients on LFD had a greater reduction in symptoms than GDA (p<0.001)	Both LFD and GDA improve symptoms for patients with IBS–LFD more than GDA
Ankersen et al., 2021 [26]	Patients were randomised to either LFD or probiotics	31 total. 15 introduced to LFD. 16 introduced to probiotics.	4 weeks	IBS Severity Scoring System – reduction of 50 points counted as a response to treatment	9/15 responded to LFD (60%)	7/16 responded to probiotics (43%)	No mean difference between LFD and probiotics in addressing IBS symptoms
Bohn et al., 2015 [27]	Patients were randomised to LFD or regular diet with advice on avoidance of foods that generally trigger IBS	75 patients (38 patients randomly assigned to LFD and 37 to regular diet).	4 weeks	IBS Severity Scoring System – reduction of 50 points counted as a response to treatment	19 responded to LFD (50%)	17 responded to regular diet (46%)	No significant difference between the two groups (p = 0.62)
Krieger-Grübel et al., 2020 [28]	13 patients were assigned to LFD and 12 patients were randomised to low Lactose Diets (LLD)	29 patients initially, however, 4 patients dropped out.	8 weeks	IBS Severity Scoring System Reduction	Significant improvement in symptoms and 13 patients reached the clinically relevant reduction of clinically relevant decrease of ≥50 points in the total IBS-SSS (p=0.002)	54.2% of patients reached the clinically pertinent reduction of ≥50 points in the total IBS-SSS (p=0.007)	No significant difference between the two groups but the LFD group reported less bloating and abdominal pain
Hustoft et al., 2017 [29]	Patients were given an LFD diet for nine weeks and then randomised to either FODMAP diet or placebo for 10 days, followed by 3 weeks of washout and then crossover	20	15 weeks	IBS Symptom Severity Scoring Index – used to measure improvement on LFD and then whether symptom relief was maintained when the fodmap diet or placebo was re-introduced	80% of patients had a continuation of symptom relief on resumption of the placebo diet	30% of patients had a continuation of symptom relief on resumption of the FODMAP diet	LFD improves symptoms in IBS
Mcintosh et al., 2016 [30]	Patients were randomised to either low or high FODMAP diet	37	3 weeks	IBS Symptom Severity Scoring Index	A higher proportion of patients 72% (13/18) showed a reduction in IBS symptoms in the low-FODMAP (p<0.009).	Only 21% (4/19) of patients in the high FODMAP group showed IBS symptom reduction.	IBS Symptoms are related to FODMAP content
Patcharatrakul et al., 2019 [31]	32 patients were randomised to “structural individual low-FODMAP dietary advice (SILFD)” and 30 patients were assigned to the “brief advice on a commonly recommended diet” (BRD)	62	4 weeks	Reduction in the IBS Symptom Severity Scoring Index by 30%	Patients with SILFD had a significantly lower score than patients with BRD - 38.5 ± 20.0 vs 53.5 ± 1.92. 18/30 (60%) patients fulfilled the responder criteria following the intervention.	The global IBS symptom severity score (VAS 0–100) post-intervention was 53.5 ± 1.92 in the BRD group. 9/30 (28%) patients fulfilled the responder criteria following BRD intervention.	LFD reduces symptoms – SILFD is more effective at reduced fodmap intake than BRD
Shepherd et al., 2008 [32]	Patients were given LFD. Then they were randomly introduced to fructose/ fructans or glucose drinks	25	2 weeks	Improvement in symptoms	14% of patients with glucose alone felt symptoms did not get better	70% of patients receiving fructose, 77% of patients receiving fructans and 79% receiving a mixture reported worsening symptoms	Dietary restriction of fructose or fructans is what improves symptoms in patients with IBS
Staudacher et al., 2017 [33]	104 patients with IBS were randomised to four different groups.	27 patients were randomised to sham diet and placebo, 26 to sham diet and probiotic, 24 to low-FODMAP diet and placebo and 27 to low-FODMAP diet and probiotic.	4 weeks	Reduction in global IBS-related symptoms and stool Bifidobacterium species concentration at follow-up.	In the low-FODMAP diet group, 29/51 (57%) patients showed symptom improvement.	20/53 (38%) patients showed symptom improvement in the sham/placebo group.	LFD reduced symptom severity in patients with IBS as compared to the placebo group, however, this difference was statistically not significant.
Ong et al., 2010 [34]	15 patients with IBS and 15 healthy patients were exposed to high and low-FODMAP diets and their symptoms were monitored	30 patients in total.	2 days	Improvement in symptoms	Symptoms better on LFD	Symptoms worse on a high FODMAP diet	Dietary FODMAPs increase hydrogen production along the GI tract and worsen symptoms in patients with IBS
Halmos et al., 2014 [35]	30 patients were assigned to a low-FODMAP diet, and eight patients were assigned to a typical Australian diet, with all food provided as frozen complete meals, but additional food lists were provided to enable the purchase of an additional FODMAP diet.	30 patients with IBS and eight healthy volunteers	9 weeks	Improvement in symptoms by using patients’ stool samples and	Patients on a low-FODMAP diet had lower gastrointestinal (GI) symptom scores (22.8, 95% confidence interval, 16.7–28.8 mm).	Patients on the Australian diet had a higher GI symptom score (44.9, 95% confidence interval, 36.6–53.1 mm).	This controlled, cross-over study showed that LDMP improved GI symptoms in patients with IBS.
Pederson et al., 2014 [36]	Patients were randomised to LFD or the probiotic *Lactobacillus rhamnosus* GG (LGG)	123	6 weeks	Improvement in IBS Symptom Severity Score or IBS Quality of Life Questionnaire	Significant reduction in IBSSS from the beginning till the end of the trial 133 ± 122 *vs* 68 ± 107	Significant reduction in IBSSS from the beginning till the end of the trial 133 ± 122 *vs* 34 ± 95	Both LFD and LGG effectively reduce symptoms in patients with IBS
Chumpiatzi et al., 2015 [37]	Patients (children) were randomised to LFD or Typical American childhood diet (TACD), 5-day washout period then switched to the alternate diet	33	9 days	GI symptom severity was measured using a 0-10 Likert scale	Fewer episodes of abdominal pain per day (1.1 ± 0.2)	More episodes of abdominal pain per day (1.7 ± 0.4)	LFD reduces abdominal pain in IBS in children
Eswaran et al., 2016 [45]	50 patients were assigned to the low-FODMAP diet, and 42 patients were assigned to the NICE diet.	92	4 weeks	Data was collected from patients using a daily questionnaire about abdominal pain, Bristol stool form, consistency, frequency and urgency.	52% of the patients on a low-FODMAP diet reported improvement in symptoms of IBS.	41% of the patients in the NICE group reported improvement in their IBS-D symptoms	Patients in the low-FODMAP diet group had a higher improvement rate in abdominal pain compared to the NICE diet group (51% versus 23%). Patients in the low-FODMAP diet had a greater reduction in abdominal pain, bloating, consistency, frequency, and urgency compared to the control group.
Goyal et al., 2021 [46]	52 patients were assigned to the low-FODMAP diet group and 49 to the traditional diet advice group.	101	4 weeks	Data was collected from patients through specific questionnaires and the response rate was defined as > 50-point reduction in IBS-SSS.	The low-FODMAP diet group had a significantly higher number of responders compared to the traditional advice group at both 4 and 16 weeks (62.7% [32/51] at 4 weeks and 52.9% [27/51] respectively. The percentage of patients compliant with a low-FODMAP diet was 93% and 64% at 4 and 16 weeks respectively.	The number of responders in the traditional group was 40.8% [20/49] at 4 weeks and 30.6% [15/49] at 16 weeks respectively.	Both groups showed significant improvement in the total IBS-SSS and IBS quality of life score, however, there was a significantly greater reduction in symptoms reduction in the low-FODMAP diet group.
Zhang et al., 2021 [47]	108 patients (54 each) were assigned to a low-FODMAP diet and BDA/NICE diet with dietary advice from a dietitian during a 20 min appointment and a menu plan to follow	100 patients (51 LFD and 49 BDA diet group) completed the study	3 weeks	Data collection was done by using questionnaires about a ≥50-point reduction in the IBS Severity Scoring System at 3 weeks and faecal samples collection.	30 of 51 (59%) patients in the LFD group achieved earlier symptomatic improvement in stool frequency and excessive wind compared to the control group.	TDA (26 of 49, 53%) patients achieved symptomatic improvement in stool frequency and excessive wind.	Both groups had reduced symptoms however LFD group patients achieved earlier symptomatic improvement compared to the BDA group and the therapeutic effect of LFD was associated with changes in the faecal microbiota and fermentation index.
Harvie et al., 2017 [48]	23 patients were assigned to a low-FODMAP diet, and 27 patients continued their habitual diet.	50 patients were enrolled in this cross-over design study	3 weeks	All patients completed the IBS SSS (IBS symptom severity scoring system, IBS quality of life (QoL) questionnaire, and a FODMAP-specific food frequency questionnaire. All patients also provided stool samples at 0, three and six months.	The low-FODMAP diet group had a significantly lower IBS SSS and increased QoL score at both 3 and 6 months.	The IBS-SSS score was high and the QoL score was low in the traditional diet group.	This cross-over study showed that a low-FODMAP diet has a positive effect on patients' QoL and symptom control.
Wilson et al., 2020 [49]	24 patients were assigned to a low-FODMAP diet and 23 patients were assigned to a sham dietary intervention	47 patients	4 weeks	Data collection was done through a questionnaire for gastrointestinal symptoms faecal microbiota and faecal short-chain fatty acids.	The adequate symptom relief was higher in the LFD/B-GOS group (16/24, 67%) at 4 weeks.	The symptom relief was lower in the control group (7/23, 30%) at 4 weeks.	Adequate symptom relief was higher in the LFD group compared to the control group (OR: 4.6, 95% CI: 1.3–15.6; P = 0.015) at 4 weeks.
Peters et al., 2016 [50]	24 patients were assigned to LFD, and 25 patients each were assigned to hypnotherapy and hypnotherapy +LFD respectively.	74 patients	6 weeks	Data collection was done by using IBS-QOL and responders were those having ≥20 mm VAS improvement in symptoms IBS-QOL	82% of patients receiving LFD showed improvement in symptoms compared to the hypnotherapy and combined intervention group.	74% of patients receiving hypnotherapy and 54% of patients receiving combined hypnotherapy and diet showed symptom improvement.	There was no difference in the number of responders with regards to primary outcome (LFD 71% vs hypnotherapy 72% vs combination 72%; p=0.67). More patients in the LFD showed symptom improvement compared to hypnotherapy and combined hypnotherapy and diet groups.
Staudacher et al., 2012 [51]	19 patients were randomised to a low-FODMAP diet and 22 patients were randomised to their habitual diet	41	4 weeks	Reduction in symptoms as reported by patients – severity score was generated by observing the mean number of days a patient had symptoms in the week	13/19 (68%) reported symptom reduction	5/22 (23%) reported symptom reduction	LFD reduced symptom severity in patients with IBS through the implications on GI microbiome are yet to be seen

The total number of patients in the RCTs was 1110. The follow-up duration ranged from 2 to 15 weeks. These studies compared the efficacy of a low-FODMAP diet to that of BDA, NICE, BRD, TACD, and a high-FODMAP diet. Most studies showed symptomatic improvement in patients on a low-FODMAP diet [8,29-37,45-49], although a few studies did not show any improvement in symptoms on a low-FODMAP diet [26-28, 50]. Most studies had smaller sample sizes (< 100 patients). A study by Zahedi et al. showed symptomatic improvement in both groups of patients, although the improvement was significantly greater in the low-FODMAP diet group [8]. Several studies have demonstrated the superiority of a low-FODMAP diet over a traditional, NICE, BDA, or high-FODMAP diet [29-31,33-37,45-50]. The studies by Ankersen et al., Bohn et al., and Grubel et al. showed no difference in symptom improvement between the two groups [26-28]. The key symptoms assessed in these studies included abdominal pain, bloating, stool consistency, frequency, and urgency using various tools such as the IBS-SSS and IBS-QoL tools.

In the study by Ankersen et al., 57% of 21 IBS patients responded to a low-FODMAP diet, and symptom remission based on an IBS-SSS lower than 175 was achieved in 8 out of 12 (67%) LFD responders and 5 out of 7 (71%) multiple probiotic responders. A significant decrease in IBS-SSS was observed in LFD and probiotic responders relative to non-responders; both responder types were adherent to the treatments. However, no significant difference in effect size between the two treatments was found; the median IBS-SSS effect sizes were -126.50 (IQR -196.75 to -76.75) for LFD responders and -130.00 (IQR -211.00 to -70.50) for probiotic responders (P>.99). The corresponding changes in QoL among LFD and probiotic responders were not significant compared with non-responders.

**Figure 7:**
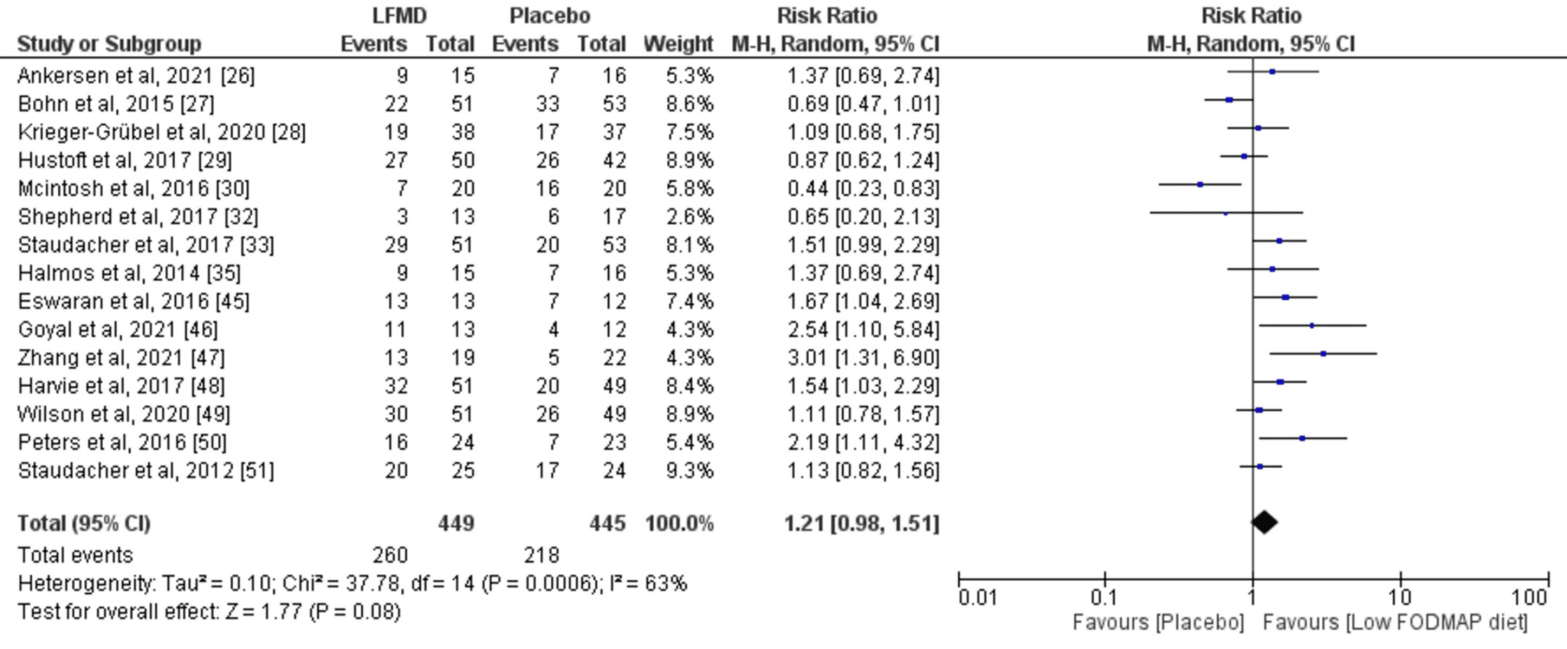
Forest plot for the randomised controlled trials included in the meta-analysis.

Krieger-Grübel et al. demonstrated that both a low-FODMAP diet and a low lactose diet produced symptom improvements based on IBS-SSS score; however, this improvement was statistically not significant [28]. The low-FODMAP diet group reported less abdominal pain and less bloating than the low-lactose diet group after eight weeks. Hustoft et al. reported that more patients found symptom relief in the low-FODMAP diet group who subsequently commenced a placebo diet of 80% than those in the high-fructose-oligosaccharides diet group which achieved 30% using the IBS Symptom Severity Scoring Index (IBS-SSS) [29]. Another study demonstrated a significant decrease in global symptom scores of 28% compared with baseline, as compared to 7% global symptom score improvement in the high FODMAP group after 3 weeks of dietary intervention [30]. A study by Patcharatrakul et al. randomised 66 patients to receive either a structural individual low-FODMAP diet (SILFD) or brief advice on a commonly recommended diet (BRD) [31]. The global IBS symptom severity score after SILFD was significantly lower than that of the BRD diet group. The symptom severity which significantly decreased from baseline after SILFD was abdominal pain, abdominal discomfort, and bloating, but not after BRD. A meta-analysis was performed including 15 studies, and a random-effects model was used for heterogeneity in the included studies. The risk ratio was 1.21 (Confidence interval: 0.98 - 1.51) and the I^2^ value was 63% supporting the efficacy of a low-FODMAP diet in patients with IBS as shown in the Forest plot (Figure 7).

**Figure 8:**
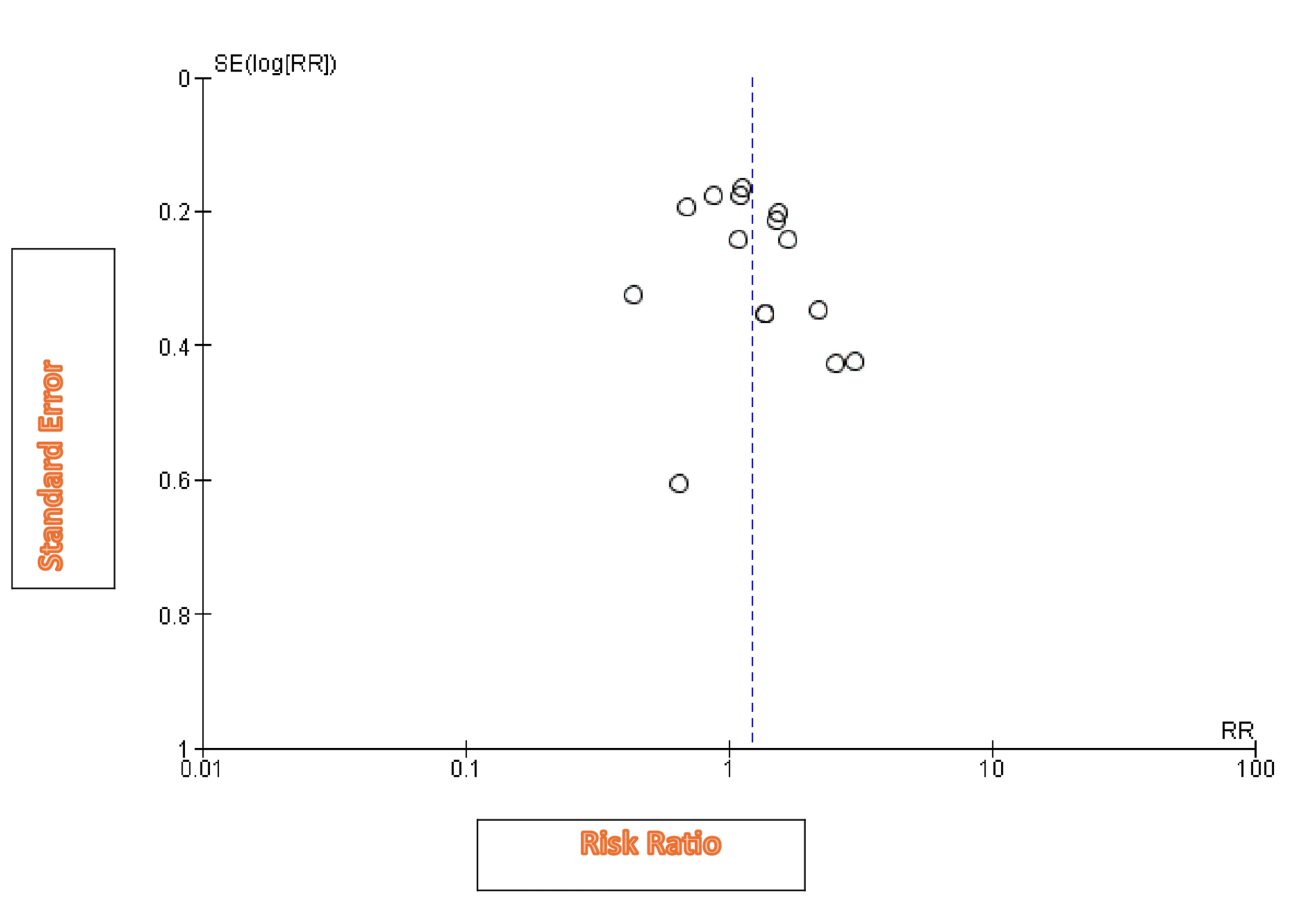
Funnel plot for the studies included in the meta-analysis.

The risk of bias assessment did not show any significant risk of bias in these studies (Figure 3). The funnel plot did not reveal any publication bias (Figure 8).

**Figure 9:**
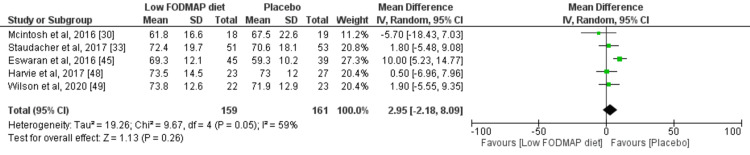
Forest plot for quality of life in the low-FODMAP diet versus placebo group. FODMAP: Fermentable Oligosaccharides, Disaccharides, Monosaccharides, and Polyols

**Figure 10:**
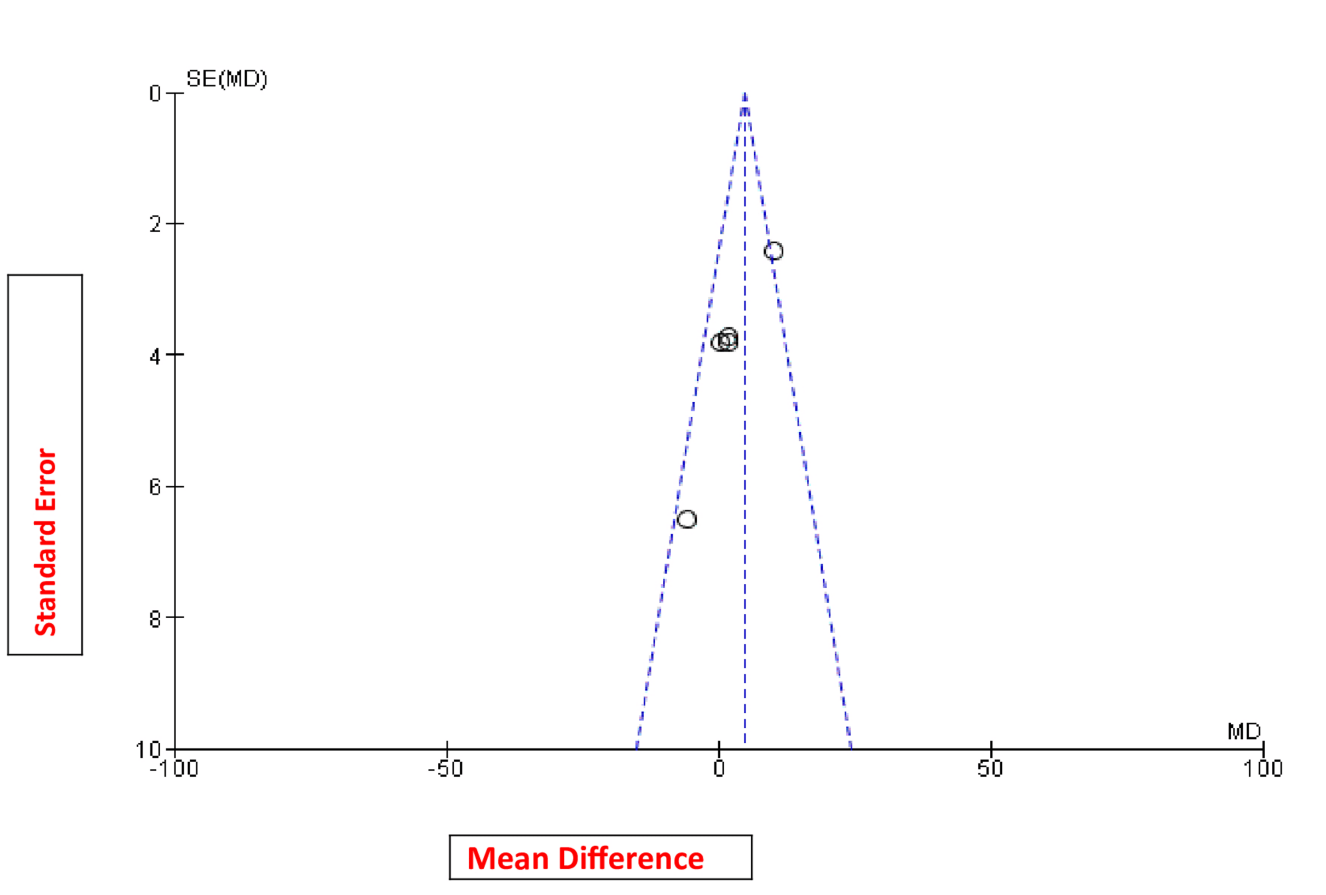
Funnel plot for studies reporting quality of life in patients included in this meta-analysis.

Five studies were included in the assessment of a low-FODMAP diet on quality of life. The total number of patients in these studies was 320 (159 in intervention group and 161 in the placebo group). The mean difference (MD) value was 2.95 and the I^2^ value was 59% when using a random effect model for heterogeneity as presented in the forest plot (Figure 9). There was no significant publication bias observed as presented in the funnel plot (Figure 10).

Six cohort studies were included in this systematic review as described in Table 4.

**Table 4:** Observational studies included in the systematic review. IBS: Irritable bowel syndrome

Author	Methodology	No. of participants	Study duration	Tools used to monitor success	Findings	Conclusion
Naseri et al., 2021 [38]	Patients were trialled on LFD with a gluten-free diet	42	6 weeks	IBS Symptom Severity Scoring (IBS-SSS) Index	IBS-SSS index decreased in 73% of patients	IBS symptoms improve with LFD and a gluten-free diet
Wong et al., 2018 [39]	Patients were initiated on LFD and monitored for symptom improvement	16	6 weeks	No tool used – subjective reduction in symptoms was the outcome	11/16 (68.8%) of patients reported improvement in symptoms	Compliance with LFD was low in patients but LFD improves symptoms
Ostgaard et al., 2012 [40]	Patients were given dietary advice and asked to answer questionnaires on their symptoms	36 IBS patients and 43 IBS patients who had received prior dietary advice two years ago along with 35 healthy control patients	2 years	FFQ, SF-NDI, IBS QOL and Birmingham IBS Score Questionnaire	After dietary advice, patients with IBS avoided FODMAP, which in turn improved their symptoms and quality of life. E.g. IBS QOL score improved – unguided IBS patients 68.5 vs guided IBS patients 75.4	Guidance on diet, which included low FODMAP intake, improves GI symptoms in patients with IBS
Mazzawi et al., 2013 [41]	Patients answered questionnaires on their IBS symptoms, then received three sessions on dietary advice and answered the questionnaires again 3-9 months later	46	9 months	IBS-QOL, SF-NDI and MoBA FFQ	Total IBS scores reduced after receiving dietary advice (p<0.001)	Dietary advice on avoidance of FODMAPs resulted in improvement in symptoms
de Roest et al., 2013 [42]	Patients were advised on LFD and monitored	90	Mean 15.7 months	General improvement in symptoms	75.6% of patients adhered to diet. 72.1% of patients reported satisfaction with symptoms	LFD is effective for patients with IBS
Pederson et al., 2014 [43]	Participants recorded their symptom severity score weekly for six weeks followed by dietary advice on LFD	19	12 weeks	IBS-SSS and IBS QOL	IBS-SSS significantly improved both during the routine recording of symptoms (278) and after the introduction of LFD (151)	Positive impact on LFD diet in patients with IBS

**Figure 11:**
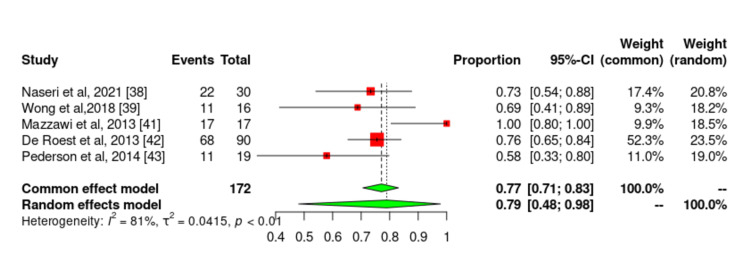
Forest plot showing values for global symptom improvement for the observational studies included in this systematic review.

A total of 327 patients were included in these studies. The follow-up duration varied from six weeks to two years, and the number of enrolled patients ranged from 19 to 114. Most studies used tools to measure symptom improvement, except for Wong et al. [39], who measured subjective improvement in patients' symptoms. All these studies showed improvement in patients’ symptoms with a low-FODMAP diet, and one study showed symptom improvement with both gluten-free and low-FODMAP diets [38]. Wong et al. reported symptom improvement with a low-FODMAP diet but low compliance among patients on this diet. The forest plot for the observational studies did not demonstrate any statistically significant improvement in patients on a low-FODMAP diet (Figure 11). A random-effects model was used to evaluate heterogeneity due to variations in the studies, as shown in the forest plot.

Discussion

IBS is one of the most common chronic gastrointestinal diseases, with a prevalence of 7-21% and is twice as common in females as compared to males in the Western world [1]. The diagnosis of IBS depends on altered bowel habits, abdominal bloating, and recurrent abdominal pain in the absence of organic bowel diseases, such as cancer or IBD. IBS is usually subdivided into four subtypes depending on the predominant symptoms: IBS-C, IBS-D, IBS-M, and unsubtyped IBS (IBS-U) [52,53]. IBS affects several aspects of patients’ lives, including impaired social function and psychological-psychiatric conditions such as anxiety and depression, and poor quality of life, and this has often been linked to poor brain and gut coordination [1,54,55].

Anxiety and depression are quite common in patients with IBS, and a study showed that anxiety is three-fold more likely in patients with IBS [55]; however, we did not assess this outcome. Research has demonstrated that diet plays a significant role in the pathophysiology of IBS, and several factors, including dietary intolerance and poor absorption of carbohydrates and fibres, contribute to this [56,57]. Food allergies occur in about 1-4% of adult patients; however, serious immunoglobulin E-mediated allergic reactions causing hives and wheezing are rare in patients with IBS [58-60]. Previous studies have shown the beneficial effects of a gluten-free diet in patients with IBS, and symptoms recurred with the introduction of gluten; however, this benefit was later confirmed to be due to the withdrawal of wheat from the diet rather than gluten [61-63].

Some studies have shown that low-FODMAP and modified NICE (mNICE) guideline diets resulted in symptomatic improvement in patients. However, a low-FODMAP diet showed significant improvement, mainly in pain and bloating, compared to the mNICE diet [45]. Most studies included in our meta-analysis also showed significant improvement in patients on a low-FODMAP diet compared with other diets. Patients with IBS may also occasionally exhibit fructose malabsorption, which can result in symptom improvement [32]. We included 36 studies in this systematic review and meta-analysis, including 11 systematic reviews, 17 RCTs, and six observational studies, which support the findings from previous trials showing symptom improvement with a low-FODMAP diet in patients with IBS. A study by Wang et al. reported global improvement in symptoms on a low-FODMAP diet compared to a sham diet, mNICE, habitual diet, high-FODMAP diet, and typical Australian diet [1]. This study also reported an improvement in stool consistency among patients on a low-FODMAP diet; however, there was no difference in quality of life between the two groups of patients. The effects of a low-FODMAP diet on quality of life vary, and a few studies have shown a positive effect [25,47,48], whereas others did not show any obvious difference [26,27]. Two RCTs assessed the effects of a low-FODMAP diet on anxiety and depression based on the Hospital Anxiety and Depression Scale (HADS), and no significant difference was noted between the two groups [6,27]. Wang et al. reported that patients on a low-FODMAP diet showed significant improvement in stool consistency and stool output. IBS is diagnosed based on recurrent abdominal pain related to changes in stool consistency and defaecation; hence, the effects of LFMD should be evaluated in patients with IBS according to the ROME III criteria [64]. Our study also showed an improvement in stool consistency and output with LFMP compared with other diet types. One study did not show any improvement in stool frequency or consistency in patients with IBS [12]. Wang et al. did not report any significant improvement in quality of patients with LFMD. Similarly, most studies included in our review did not report quality of life as an outcome; however, a few studies reported improvements in the quality of life of patients with LFMD [14,41,47]. Mazzawi et al. (2013) reported significant improvement in quality of life in IBS patients with an LFMD [41]. A few studies had a very high percentage of patients dropping out, and symptom improvement, lack of compliance, and lack of motivation were common factors responsible for this dropout.

**Figure 12:**
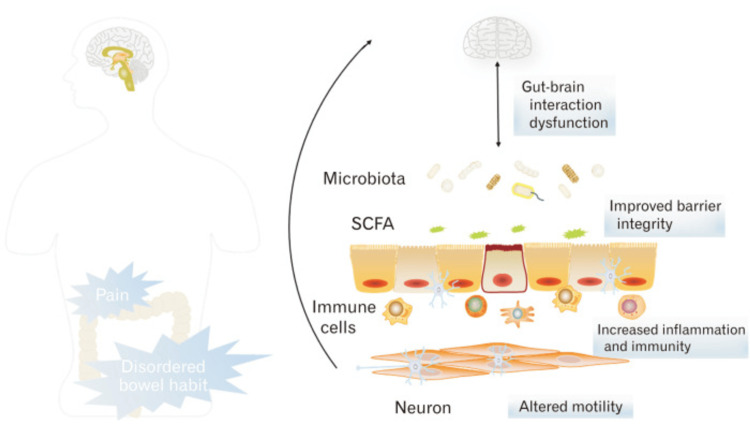
The role of SCFAs in the pathogenesis of IBS by modulating the modulate intestinal epithelial immunity and inflammation, maintaining gut barrier integrity, altering gut motility, and acting as part of the microbiota-gut-brain axis. Permission was obtained from the authors to reproduce this image [67]. SCFA: Short-chain fatty acid; IBS: Irritable bowel syndrome

The gut microbiota plays a pivotal role in the regulation of endocrine, metabolic and immune functions by modulating multiple neurochemical pathways through the highly interconnected gut-brain axis [65]. Although the gut microbiota colonizes several other cavities in the human body such as pulmonary, oral and nasal however their highest density is found in the digestive system by existing in a symbiotic relationship with the host [65,66]. Recent studies have suggested that short-chain fatty acids (SCFAs), bile acids and amino acid-derived metabolites that are derived from gut microbiota may play a role in the development of IBS [67]. SCFAs are the end product of non-absorbed carbohydrates in the intestine and have shown promising effects against various health conditions such as diabetes, inflammation, depression and other immune-deficient conditions [68]. A recent study demonstrated a lower microbiota diversity in IBS patients compared to controls and an increase in Firmicutes and a depletion in Bacteroidetes was observed in patients with IBS-C and IBS-M respectively [69]. Another study showed a significantly higher level of acetate, propionate, and total SCFAs in patients with IBS compared to controls, which correlated with the severity of symptoms [70]. Additionally, SCFAs also have effects on gut mobility regulation, microbiota-gut-brain axis and intestinal barrier integrity. Figure 12 depicts the various mechanisms by which SCFAs play a role in the pathogenesis of IBS.

IBS affected the quality of life in most patients, and LFMD showed improvement in quality of life in this group, although this may not be statistically significant. This is associated with significant costs to both employees and employers, and effective therapies to manage and treat this condition to alleviate the financial and societal burden associated with it. It is also important to highlight that undue excessive restriction and exclusion of contents from the LFMD diet can negatively affect adherence in patients with IBS [1,35]. Most patients with IBS can easily identify the type of food they have been randomised to, making the randomisation and blinding processes difficult. A low-FODMAP diet has several important physiological effects, such as increasing stool bulk, enhancing calcium absorption, modulating immune function, and decreasing the levels of triacylglycerols, serum cholesterol, and phospholipids. As a result, concerns about nutritional insufficiency have been raised. Most of these studies had a shorter duration; however, longer-duration studies are recommended to assess both the positive and negative effects of LFMD. Although longer term studies are recommended, however, the effects of dietary restriction in patients may result in nutritional deficiencies and heterogeneity is another major issue encountered in most of these studies.

Strengths & Limitations

The main strength of this review is that we included 36 studies, and most studies had a low risk of bias. Additionally, this review compared the efficacy of the LFMD diet with various other diets, and we included 15 studies in our meta-analysis. The weakness of this study is that most observational studies did not provide sufficient data to perform a meta-analysis for the observational studies. Additionally, most studies did not provide sufficient numerical data on quality of life and the effect of LFMD on anxiety. Further trials focusing on the effects of LFMD on quality of life and anxiety in patients with IBS would be useful. Most studies included in our review had a shorter follow-up duration, except for a few studies which is another limitation of our review.

## Conclusions

In conclusion, a low-FODMAP diet has positive effects on global symptom improvement in patients with IBS. It also improves the quality of life of IBS patients and reduces the frequency of symptoms. Additionally, it significantly improves abdominal pain, bloating, stool consistency, and frequency in IBS patients. Our current study showed numerical improvement in quality of life in patients with IBS. However, this effect was statistically not significant. Further studies evaluating the role of a low-FODMAP diet on the quality of life and anxiety are recommended. Dietitians' support is crucial in managing patients with IBS to ensure their nutritional requirement is met while adhering to a certain diet. These effects were more pronounced in patients with IBS-D than in those with other subtypes of IBS.
